# Phase boundary of hot dense fluid hydrogen

**DOI:** 10.1038/srep16560

**Published:** 2015-11-09

**Authors:** Kenji Ohta, Kota Ichimaru, Mari Einaga, Sho Kawaguchi, Katsuya Shimizu, Takahiro Matsuoka, Naohisa Hirao, Yasuo Ohishi

**Affiliations:** 1 Center for Science and Technology under Extreme Conditions, Osaka University, Toyonaka, Osaka 560-8531, Japan; 2 Department of Earth and Planetary Sciences, Tokyo Institute of Technology, Meguro, Tokyo 152-8551, Japan; 3 Japan Synchrotron Radiation Research Institute, Sayo, Hyogo 679-5198, Japan

## Abstract

We investigated the phase transformation of hot dense fluid hydrogen using static high-pressure laser-heating experiments in a laser-heated diamond anvil cell. The results show anomalies in the heating efficiency that are likely to be attributed to the phase transition from a diatomic to monoatomic fluid hydrogen (plasma phase transition) in the pressure range between 82 and 106 GPa. This study imposes tighter constraints on the location of the hydrogen plasma phase transition boundary and suggests higher critical point than that predicted by the theoretical calculations.

Condensed hydrogen displays several phase changes at high pressures (*P*) and temperatures (*T*) (ref. 1), and it may undergo solid metallic phase with the high-*T* superconducting nature of certain conditions^2^. As the physical properties of condensed hydrogen have been accepted to be critically important not only for condensed matter physics but also for astrophysics, there has been a great deal of experimental and theoretical studies on hydrogen. The phase relation and density of hydrogen are key issues for the interpretation of the internal structure of gas giants such as Jupiter and Saturn^3^. It is thought that their observed strong symmetric dipole magnetic fields are generated from the convective motion of conductive dense hydrogen that could be monoatomic fluid hydrogen dissociated from the diatomic molecular fluid phase–the so called plasma phase transition–at a certain depth of the gas planet’s envelope. Hence, the nature of the plasma phase transition of hydrogen (and deuterium) has been extensively investigated by experiments^4^,^5^,^6^,^7^,^8^,^9^ and theoretical calculations^10^,^11^,^12^,^13^,^14^,^15^,^16^,^17^. However, the location of the plasma phase transition boundary of dense hydrogen is still a subject of argument. *Dzyabura et al.*^8^ observed a plasma phase transition in a laser-heating experiment with a laser-heated diamond anvil cell (LHDAC), but the pressure range was limited to just around 120 GPa. Since the plasma phase transition boundary would have a steep negative Clapeyron slope, there is a need to extend the pressure range of the experiments to produce a tighter constraint on the phase boundary.

Therefore, we revisit the use of an LHDAC for the determination of the hydrogen plasma phase transition boundary in the pressure range between 82 and 106 GPa. As reported by *Dzyabura et al.*^8^, a criterion for the hydrogen plasma phase transition is the existence of an anomaly in the laser-heating efficiency. When the sample undergoes a phase transition, a change in the slope of the temperature–laser-power curve can be seen due to latent heat of the transition and/or due to increase in conductivity and reflectivity^18^. The *P-T* conditions of the plasma phase transition determined in this study appear to be correlated with some previous theoretical predictions^10^,^11^,^12^,^13^,^15^ and experimental results^8^.

## Results

**Figure 1:**
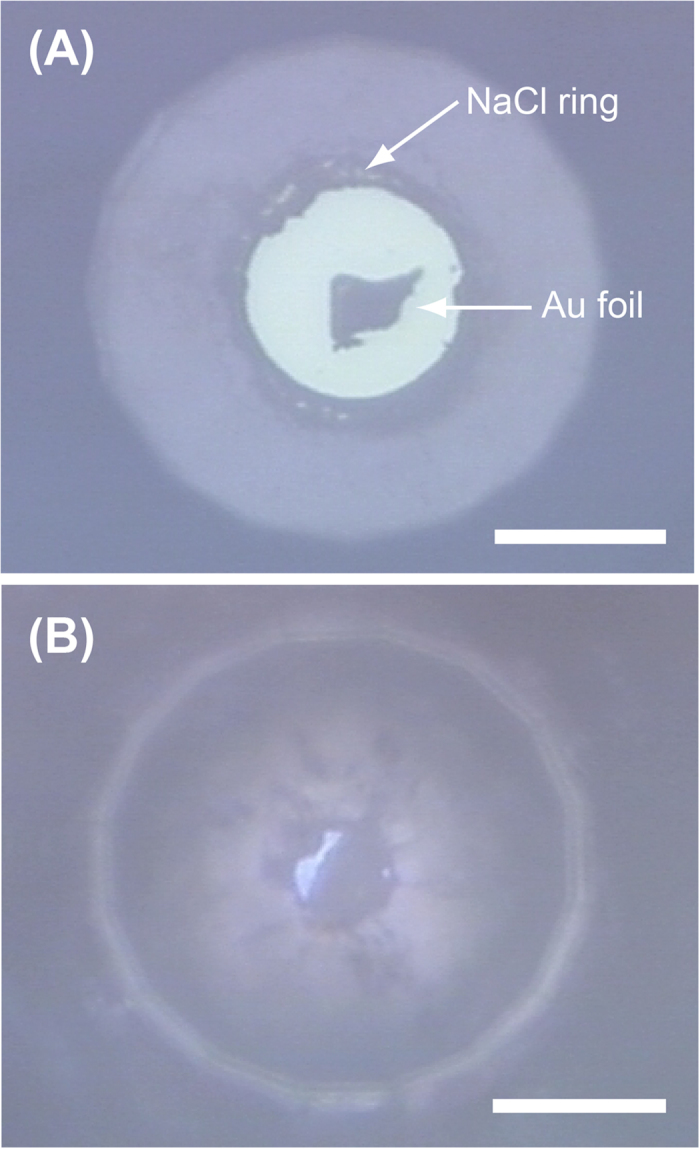
Photographs of the sample chamber (**A**) before hydrogen injection at 1 bar and (**B**) after injection at 106 GPa and room temperature. The diamond anvils are protected from hydrogen penetration by thin Ti layer. The wall of rhenium gasket is covered by NaCl, which prevent hydrogen diffusion into rhenium gasket. Gold foil was served as laser absorber for laser heating. White scale bars indicate 40 μm.

**Figure 2:**
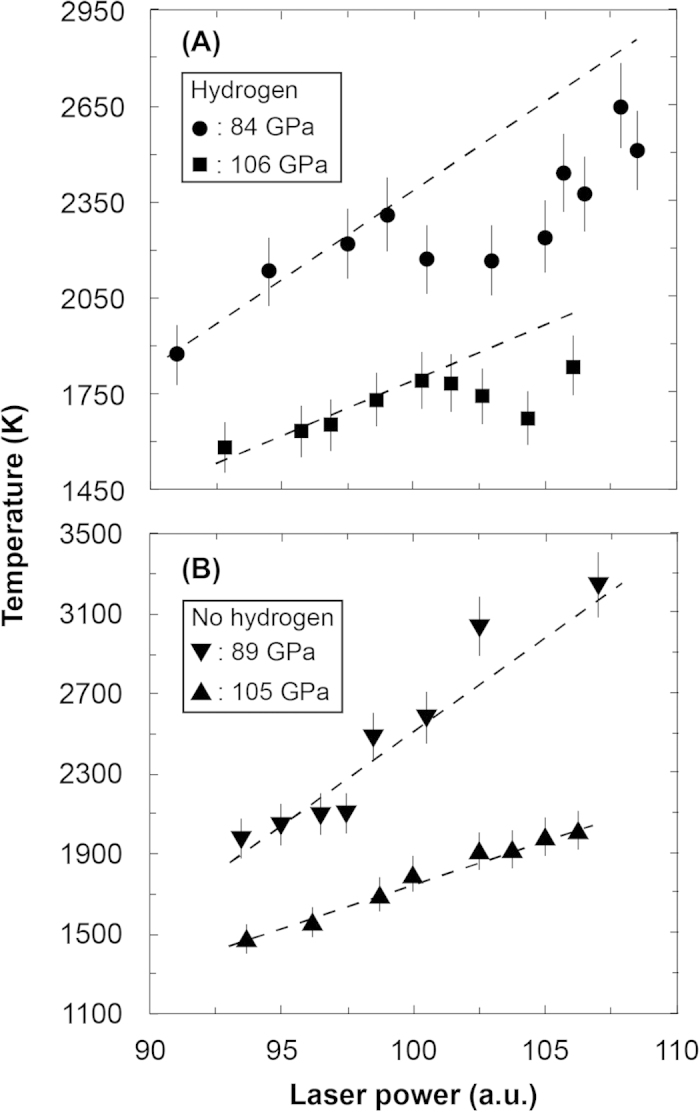
Representative laser power versus sample temperature curves. (**A**) The curves obtained at 84 GPa (circles) and 106 GPa (squares) when a sample chamber was filled by hydrogen and gold foil. (**B**) The curves found at 89 and 105 GPa when no hydrogen was loaded in the sample chambers. The broken straight lines are guide to the eye.

We carried out three sets of laser-heating experiments on hydrogen using different LHDACs to determine the plasma phase transition boundary. In the sample chamber, a gold foil that served as an infrared laser absorber was loaded together with hydrogen and compressed to the target pressure at 300 K (Fig. 1). At the target pressures, hydrogen and gold foil were heated by using a couple of continuous-mode infrared fibre lasers. The hydrogen also serves as a thermal insulator of the gold foil against the diamond anvil during laser heating. Since hydrogen is extremely diffusive and reactive element, high *P-T* experiment for hydrogen is challenging. Although rhenium is commonly used as a gasket material in a LHDAC, rhenium readily reacts with hydrogen to produce rhenium hydride. Additionally, above 500 K in the presence of pressurized H_2_, rhenium (or rhenium hydride) typically loses its strength and its performance is degraded due to hydrogen brittleness, which often induces hydrogen sample loss. Hydrogen also enters into the diamond anvils and often wrecks them at high *P-T* conditions. In order to circumvent these problems derived from highly diffusive and reactive hydrogen, we employed composite gaskets made of compressed sodium chloride (NaCl) and rhenium (Fig. 1). A ring of NaCl separates the hydrogen sample from the rhenium gasket to prevent gasket embrittlement and hydridation. The surface of the diamond anvils was coated with about 50 nm thick Ti layer, which prevents the hydrogen from escaping into the anvils. The new techniques of the composite gasket made by rhenium and NaCl and the Ti coating on the surface of diamond anvils successfully avoid hydrogen escape from the sample chamber up to 2650 K. The presence of hydrogen in the sample chamber was confirmed by the detection of the Raman spectrum of hydrogen vibrational mode before and after laser heating for all the runs. Relationships between the sample temperature and the laser output power were obtained in each heating cycle (Fig. 2).

**Figure 3:**
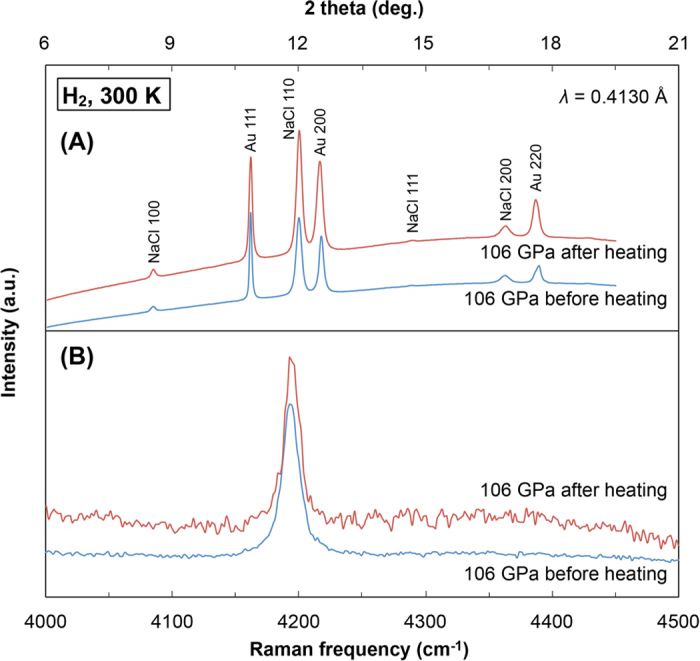
(**A**) XRD spectra of Au and NaCl and (**B**) Raman spectra of hydrogen vibron mode obtained at 106 GPa and room temperature before and after laser heating.

In the first run, the laser-heating experiment was conducted at 106 GPa. By increasing the laser power, the sample temperature monotonically increased up to 1790 K; it then decreased during a few more steps of increased power, and it again increased to the maximum temperature of 1835 K (Fig. 2A). X-ray diffraction spectra of the gold foil and Raman spectra of the hydrogen were collected before and after the laser-heating experiment. Both of the spectra were not changed by laser heating, implying that no chemical reactions occurred in the sample chamber (Fig. 3). A similar experiment was performed after a decompression of the same hydrogen sample to 92 GPa, and we also observed a heating efficiency change at around 2220 K. This behaviour has been repeatedly observed in laser-heating experiments on hydrogen, and the anomaly (plateau) in the temperature vs laser-power curve was interpreted as the sign of melting^19^ and the plasma phase transition^8^. In the second set of experiments, the hydrogen sample was compressed to 82 GPa at 300 K and heated. As shown in the results of the first run, an anomaly was detected at 2450 K in the temperature–laser-power curve. After the heating experiment, we slightly increased the sample pressure to 84 GPa and conducted a similar heating experiment (Fig. 2A). The local peak temperature was 2310 K, which is lower than that obtained at 82 GPa. A third experiment performed at 85 GPa corroborated the existence of the local maximum temperature of 2450 K, which is in good agreement with the former experiments.

The uncertainty of the measured temperatures calibrated from thermal radiation of the sample and gold foil was within 5%. Hence the changes in slope of the power-temperature curves are not hidden inside the error bar (Fig. 2). According to the modelling of heat transfer in a LHDAC by *Geballe and Jeanloz*^18^, latent heat due to a phase transition is unlikely the only source of the observed change in the laser-heating efficiency, regardless of whether pulsed or continuous lasers were used. Rapid increases in thermal conductivity and reflectivity of the sample also contribute to cause the anomaly in heating efficiency during the laser heating. The plasma phase transition in hydrogen may be strongly related to insulator-metal transition, and thus the increases in conductivity and reflectivity across the plasma phase transition are highly expected^1^. Here we repeatedly observed the plateau-like structure in the temperature vs laser-power curves as shown in Fig. 2A, which could be the strong evidence for the plasma phase transition. Extrinsic causes such as a chemical reaction in the sample chamber and change in surface roughness of gold laser absorber could induce the plateau like feature. However, we confirmed no chemical reaction in the sample chamber and no change in appearance of surface roughness of gold foil after the laser-heating experiments (Fig. 3).

To compare the results of the hydrogen laser-heating experiments, we performed similar experiments at 89 and 105 GPa, but we loaded the gold foil without hydrogen injection into the sample chamber of the LHDACs. In the control experiments, the gold foil was sandwiched by NaCl for thermal insulation. The obtained results show no anomaly in heating efficiency during both the heating and cooling cycles (Fig. 2B), whereas we detected the plateau-like feature at 106 GPa and 1790 K in the first run and at 84 GPa and 2310 K in the second run of the hydrogen laser-heating experiments (Fig. 2A). These results in the control experiments support our argument that the changes in the heating efficiency observed are due to the plasma phase change of hydrogen under high pressure and temperature conditions. The melting temperature of pure gold is more than 3500 K above 85 GPa (ref. 20).

## Discussion

**Figure 4:**
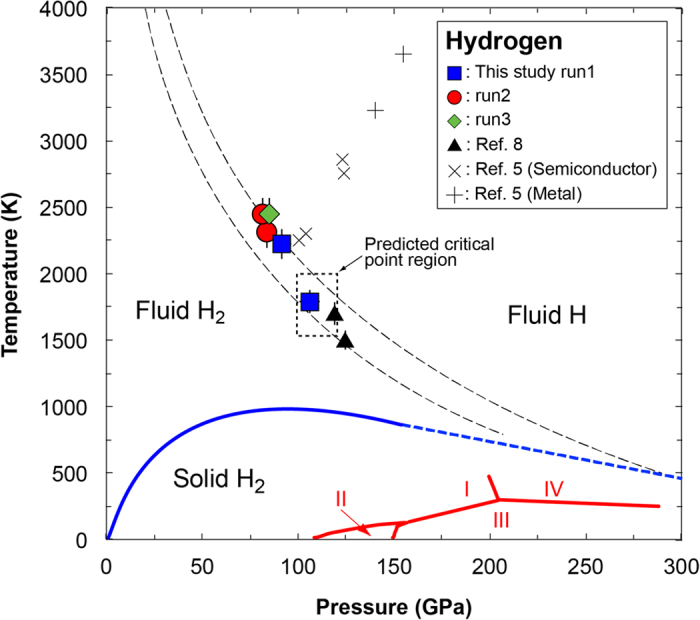
Phase diagram of dense hydrogen. For the plasma phase transition boundary, we show a range of curves resulting from reported theoretical calculations^10^,^11^,^12^,^13^,^15^ by black broken lines. The predicted position of the critical point is indicated by dotted box^13^. Melting curve and phase boundaries of solid hydrogen are taken from the literature^8^. Cross and plus symbols represent *P-T* conditions at which electrical conductivities were measured under shock compression^5^.

Here we determined the plasma phase transition temperature from the local peak temperature in the temperature–laser-power curves obtained in this study (Figs 2 and 4). *Dzyabura et al.*^8^ recently conducted similar laser heating experiments at 119 and 125 GPa and high temperatures in a LHDAC, and presented the evidence of the plasma phase transition, which is in good agreement with our data (Fig. 4). These static high *P-T* experiments confirm that the plasma phase transition of hydrogen has a steep negative Clapeyron slope as many theoretical calculations predicted. *In-situ* high *P-T* Raman spectroscopy on hydrogen has been performed up to 140 GPa and 1500 K, but it did not show any evidence of the plasma phase transition of hydrogen^21^, which also imposes strong constraints on the location of the plasma phase transition boundary.

Dynamic compression studies enable to cover much higher *P-T* conditions corresponding to the internal *P-T* conditions of gas giants to investigate the nature of hot dense hydrogen. Several attempts were performed to detect phase transitions of hydrogen (and deuterium) at high *P-T* conditions from changes in electrical resistivity, density and optical reflectance^4^,^5^,^6^,^7^,^9^,^22^. Earlier shock compression studies showed gradual decrease in electrical resistivity with compression and minimum hydrogen resistivity above 140 GPa (refs. 4,5) (Fig. 4). Recent study for shocked dense liquid deuterium^9^ also reported the experimental evidence for the plasma phase transition. They found that the deuterium sample became opaque at ~120 to 150 GPa and high temperatures (~1100 to 1600 K), which was interpreted as band gap closing to ~2.1 eV. These *P-T* points are in good agreement with the suggested boundary of the plasma phase transition from earlier and this studies^8^,^10^,^11^,^12^,^13^,^15^.

The nature of hydrogen plasma phase was also investigated via theoretical calculations. Earlier computations using density functional theory (DFT)^10^,^11^,^12^,^13^,^15^ predicted the *P-T* conditions of the plasma phase transition that are in good agreement with *Dzyabura et al.*^8^ and the present study (Fig. 4). However, recent DFT calculation including nuclear effects^14^ and a diffusion Monte Carlo calculation^17^ made an upward revision of the *P-T* conditions of the plasma phase transition, which resulted an increase of the predicted plasma phase transitions pressure of more than 100 GPa. Present experimental results along with *Dzyabura et al.*^8^ would help to resolve these large discrepancies in the theoretical predictions (Fig. 4).

The location of the critical point is important for modelling the internal structure of gas giants. Abrupt changes in the density and degree of ionization are expected to occur below the critical point, whereas the changes will be gradual above the critical point. The critical point of dense hydrogen is first predicted to occur at around 60 GPa and 15300 K where is much higher than the predicted temperature of Jupiter’s interior at the equivalent pressure^23^, while the other calculation employing DFT suggested lower *P-T* critical point condition of 100 GPa and 1500–2000 K (ref. 13)(Fig. 4). We performed the laser-heating experiments both above and below the predicted critical point^13^, and observed similar behaviours in the temperature–laser-power curves (Fig. 2). Our results therefore suggest that the critical point lies above 2450 K.

In conclusion, we carried out the laser-heating experiments on dense hydrogen and found anomalies in the heating efficiency of hydrogen in contact with a gold laser absorber (Fig. 2A). XRD measurements and Raman spectroscopy performed before and after the laser-heating experiments showed no evidence of chemical reaction between hydrogen and surrounding materials (Fig. 3). And, we did not observe such anomaly in heating efficiency in the control experiments that employed similar cell configurations but no hydrogen loading (Fig. 2B). We therefore determined the temperatures of the plasma phase transition that resulted in local maximum temperatures of the temperature–laser-power curves, and plotted them as functions of the pressure (Fig. 4). The results are in good agreement with the previously suggested plasma phase transition boundary^8^,^10^,^11^,^12^,^13^,^15^. In addition, our results suggest higher critical point than previous predictions^13^. However, the experimental technique employed both by *Dzyabura et al.*^8^ and in this study shows an indirect evidence of the occurrence of the plasma phase transition. A significant change in electrical and thermal conductivity and Raman spectrum is a definitive sign of phase transition of molecular hydrogen^4^,^5^,^21^,^24^. Nevertheless, *in-situ* static high *P-T* probing of hydrogen phase change is still very challenging because of high diffusivity and reactivity of hot hydrogen. Our new hydrogen sample configuration technique succeeded to keep hydrogen up to 2650 K at static high pressures in a LHDAC, and the technique would help to detect the critical point and to clarify the nature of the hydrogen plasma phase transition.

## Methods

All experiments were carried out using the LHDAC with 120 μm culet beveled diamond anvils. Hydrogen was loaded in the LHDAC using a cryogenic hydrogen loading system at the Center for Science and Technology under Extreme Conditions, Osaka University^25^. We newly employed the composite gaskets made of compressed sodium chloride (NaCl) and rhenium (Fig. 1). The surface of the diamond anvils was coated with about 50 nm thickness of Ti film to prevents the hydrogen from escaping into the anvils. We place a gold foil into the sample chamber with a composite gasket before hydrogen injection. After hydrogen injection into the sample chamber of the LHDAC, the sample is first compressed to the pressure of interest at room temperature. We also prepared a sample configuration composed of the gold foil and NaCl without the hydrogen for the control experiments at 89 and 105 GPa.

Laser-heating experiments on the compressed hydrogen were carried out at the BL10XU of SPring-8. Double-sided heating with a couple of 100 W continuous fibre lasers was employed to heat the sample, which reduced the axial temperature gradient in the sample chamber. The diameter of the heated area was approximately 40 µm. Hydrogen and NaCl act as thermal insulators against the diamond anvil and rhenium gasket during laser heating. We collected thermal radiation from the sample and analysed it in a wavelength between 550 and 750 nm to convert the temperature in accordance with the Planck’s black body radiation law^26^. This method allows us to calibrate sample temperatures above 1300 K. The temperature variations across the laser-heating spot were less than 5%. On the basis of the estimation of the uncertainty of measured temperature, the error bars are shown in Fig. 2. Because the contacting hydrogen could have the same temperature as the gold foil laser absorber, we consider the calibrated temperature as the temperature of the hydrogen (see Fig. S2 of ref. 21). The pressures were determined from the Raman spectra of the diamond anvils at room temperature before and after the laser heating^27^. The uncertainty in the pressure determination was less than 5 GPa at the highest pressure. The pressure difference before and after the heating experiment was about 1 GPa. Thermal pressures due to laser heating were estimated to be about 2 GPa as reported by *Dewaele et al.*^28^.

## Additional Information

**How to cite this article**: Ohta, K. *et al.* Phase boundary of hot dense fluid hydrogen. *Sci. Rep.* **5**, 16560; doi: 10.1038/srep16560 (2015).
